# Outcome of carcinoid heart syndrome in patients enrolled in the SwissNet cohort

**DOI:** 10.1186/s12885-023-10739-z

**Published:** 2023-04-13

**Authors:** Eva Grundmann, Alessandra Curioni-Fontecedro, Emanuel Christ, Alexander R. Siebenhüner

**Affiliations:** 1 Department of Medical Oncology and Hematology, University Hospital Zurich and University Zurich, Rämistrasse 100, Zurich, CH-8091 Switzerland; 2 Department of Endocrinology, Diabetes, and Metabolism, Basel University Hospital, University of Basel, Basel, Switzerland; 3 Clinic of Medical Oncology and Hematology, Hirslanden Zurich AG, Witellikerstrasse 40, Zurich, CH-8032 Switzerland

**Keywords:** CHD, Echocardiography, Hedinger syndrome, NET, Neuroendocrine tumors, Neuroendocrine treatment, Overall survival

## Abstract

**Background:**

Carcinoid heart disease is a rare disease which develops in patients with functional neuroendocrine tumors in an advanced tumor state. Patients diagnosed with carcinoid heart disease have a poor longtime prognosis with respect to morbidity and mortality and long-term data on patient outcomes are lacking.

**Methods and results:**

In this retrospective study, we analyzed outcomes of 23 patients with carcinoid heart disease enrolled into the SwissNet database. We observed that early diagnosis with echocardiographic surveillance of carcinoid heart disease during the course of the neuroendocrine tumor disease was beneficial to overall survival of patients.

**Conclusion:**

Through nationwide patient enrollment, the SwissNet registry is a powerful data tool to identify, follow-up and evaluate long-term patient outcomes in patients with rare neuroendocrine tumor driven pathologies including carcinoid heart syndrome with observational methods enabling better therapy optimization to improve patient`s long-term perspectives and survival. In line with the current ESMO recommendations, our data proposes that heart echocardiography should be included as part of the general physical assessment in patients with newly diagnosed NET.

## Highlights

CHD is associated with poor prognosis

Early CHD detection prolongs survival

Echo as baseline prior NET therapy is recommended

## Introduction

Carcinoid heart disease (CHD) is a major cause of increased morbidity and mortality in patients diagnosed with neuroendocrine tumors (NET) [1–3]. CHD, a rare valvular disease, is characterized by plaque deposition at the endocardial surfaces of the right-sided heart valves, papillary muscles, and chordae tendineae and develops from NETS causing carcinoid syndrome [2, 4, 5]. Those tumors arise most frequently from the gastrointestinal tract and bronchopulmonary system and are slow growing tumors [2, 6–8]. They typically present as indolent and trigger the release of vasoactive substances e.g. serotonin which are deactivated by the liver via the first pass effect [2, 6–8]. Patients often present as asymptomatic until the NET metastasizes into the liver leading and interferes with the degradation of the vasoactive substances [2, 4, 5, 9, 10]. Thereby the systemic increase of serotonin leads to carcinoid syndrome, a pattern of symptoms including abdominal pain in 35%, diarrhea in approximately 60–80% and flushing in 90% of patients, respectively, with a NET at time of diagnosis [11]. Large amounts of vasoactive substances such as serotonin, tachykinins, and prostaglandins circulate into the right side of the heart leading to CHD [2, 4, 5, 9, 10]. Serotonin excess is believed to initiate heart fibrosis, correlating with high levels of serotonin metabolites in the 24 h urine of patients with CHD and serotonin receptor expression on their cardiac valves [12–14]. Other vasoactive factors might trigger CHD as well as some patients experience progressive CHD despite having low serotonin levels [2]. The chronic exposure to these substances as well as transforming growth factor-β is believed to induce an initial endocardial injury followed by plaque deposition at the endocardial surfaces of the right-sided heart valves, papillary muscles, and chordae tendineae [2, 4, 5]. The plaque, consisting of fibroblasts, smooth muscle cells, and extracellular matrix, is often detected downstream, at the ventricular aspect of the TV and the pulmonary arterial side of the PV [2, 5, 10]. The left side of the heart is usually spared from endocardial injury likely due to the fact that the lung metabolizes the vast majority of those substances [2, 4]. Left-sided lesions were reported in only up to 15% of patients with CHD and usually occur in patients with patent foramen ovale and primary bronchopulmonary carcinoid disease [2, 4, 5, 10]. Up to 70% of patients with a NET causing carcinoid syndrome will eventually develop CHD which is associated with a poor long-term prognosis with an estimated 3-year survival rate of 31% vs 68% seen in patients with a NET causing carcinoid syndrome without heart involvement [1–3]. Patients with CHD are initially asymptomatic and clinical manifestations become more pronounced along with right-sided heart involvement which includes all signs of progressive heart failure such as exertional dyspnea, fatigue, systolic murmur, ascites, jugular venous pressure elevation, weight gain, right upper abdominal pain and peripheral edema [4, 9, 15, 16]. CHD is usually diagnosed on the basis of right ventricle (RV) thickening and reduced mobility/or retraction of the tricuspid (TV) and pulmonary valve (PV) via 2-dimensional transthoracic echocardiography (TTE) [16–18]. TV regurgitation is the most prominent valve pathology detected in up to 92–100% of patients with CHD [19]. In the majority of patients the leaflets are fixed in a half-open position leading to TV stenosis and up to 88% of patients can present with PV pathology (regurgitation or stenosis) [9, 19]. Therefore, it is recommended that patients with carcinoid syndrome with documented cardiac involvement receive a TTE screening every 6 months and those without cardiac pathologies once per year [20]. Biochemical markers used to diagnose CHD include NT-proBNP, a hormone released by the atria and ventricles in response to stretching from volume or pressure overload, 5-HIAA, a serotonin metabolite and chromogranin A, a less sensitive and specific protein secreted by neuroendocrine tumors, can be elevated in the serum of patients with CHD and rising levels correlate well with disease progression [2, 4, 17, 20]. Patients with CHD need a multidisciplinary treatment management including controlling of progressive heart failure, treatment of systemic malignancy and neuroendocrine abnormalities, surgical intervention to correct right-sided valvular derangements and if feasible surgical downsizing/ resection, local radiation (selective internal radio therapy [SIRT]) and transarterial embolization of liver metastases [2, 4, 11]. Symptom management of right-sided heart failure include careful administration of loop thiazide and/ or aldosterone and salt and volume intake restrictions [2, 4, 10]. The only definite intervention relieving CHD symptoms is TV and/ or PV valve replacement which increases the median survival to 6–11 years [2, 4, 5, 9–11, 16, 21]. Until today, limited data on patient outcomes with NET and CHD is available as most data are derived from small case series [2, 22]. Therefore, we analyzed the frequency and outcomes of patients with NET and CHD enrolled into the SwissNet database with close to 2000 patients by the end of 2019 to check whether early diagnostical and therapeutical interventions decreases the emergence of CHD diagnosis in patients with NET.

## Materials and methods

This is a retrospective patient chart review of adult patients with confirmed NET and CHD enrolled into the SwissNET registry (registered under NCT01039922; https://clinicaltrials.gov/ct2/show/NCT01039922). The SwissNet registry was founded in 2005 and is a national and interdisciplinary consortium which prospectively included patients with a NET since 2008 [23, 24]. The SwissNET registry aims at optimizing therapy strategies on the basis of real-world patient data. The scientific committee of SwissNet approved the study, the project has received a positive ethical approval (cantonal ethics Berne, committee number: 395/14) and all patients gave informed consent to publication and data sharing in accordance to the Declaration of Helsinki [25].

For all patients with functional tumors (*n* = 207), echocardiography had been carried out to screen for CHD, following institutional routine. The data lock point was 31^st^ of August 2020. Calculations and visualization of patient data were performed using Excel and R. Survival analysis was prepared according to Kishore et al. [26]. Briefly, patients at risk were evaluated in a 6-month interval. Patients who were still alive at the time of data lock-point, were only evaluated for time-points which matched their follow-up status and were excluded from later time-points of interest. Survival was analyzed for a time span of 5 years. Patient survival was plotted according to the Kaplan–Meier method and comparisons of death rates between subgroups were tested with the log-rank test. Demographics and clinical parameters between patient groups were compared using Fisher exact test. For the comparison of patient outcomes of this retrospective study to previously described data, a systematic literature search in PubMed using terms for CHD was conducted. Eligible articles had to describe studies that included patients with carcinoid syndrome and reported data on predefined CHD outcomes.

## Results

**Fig. 1:**
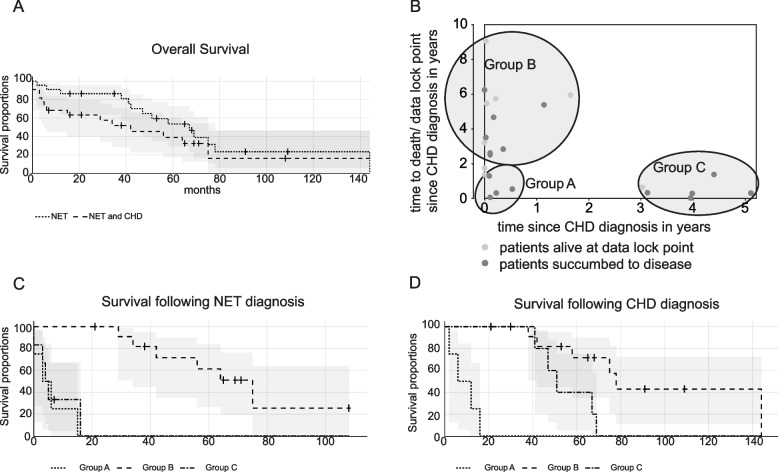
Patient data derived from SwissNET and 23 patient diagnosed with NET and CHD were included. **A** Survival proportions of patients following initial NET diagnosis and survival proportions of patients following initial CHD diagnosis after NET diagnosis. **B** Patient cluster with respect to time to CHD diagnosis and survival. **C** Survival proportions of patient groups since NET and CHD diagnosis (Group A; Group B and Group C); Confidence interval 95%—grey area; Log-rank test Group A vs Group B: *p* =  < 0.005 and Group B vs Group C: *p* =  < 0.005. **D** Survival proportions of patients with NET; Confidence interval 95%—grey area; Log-rank test Group A vs Group B: *p* =  < 0.005; Group B vs Group C *p* = 0.03. For **A**, **C** and **D** please refer to Materials and methods section for patients at risk calculations. + ; patient was censored

At 31^st^ December of 2019, 1811 patients with NET were enrolled into the SwissNET database. In the majority of patients (*n* = 1251, 68%), the tumor was characterized as non-functional, 207 patients (11%) were diagnosed with a functional tumor and 395 patients (21%) where diagnosed with a neoplasm where it was not reported whether the tumor was functional or not. The majority of patients (*n* = 108, 52%) with a functional tumor presented with a NET causing carcinoid syndrome. The frequency of patients with a carcinoid syndrome among patients with NET in the SwissNET database was 5.8%. Of those 108 patients, 23 patients were diagnosed with CHD which was confirmed by echocardiography, accounting for a CHD frequency of 21% among patients with NET causing carcinoid syndrome. Tables 1, 2, 3 and 4 summarize characteristics of patients with CHD. With respect to time point of initial NET diagnosis, 63% of patients with CHD (16 out of 23 patients) were deceased with a corrected 1, 3 and 5-year mortality rate of 9% (2 patients deceased); 20% (4 patients deceased) and 53% (10 patients deceased), respectively at time of reporting (Fig. 1A). The median time to CHD diagnoses was 3 months (interquartile range [iqr] 28 months) with 8 patients being diagnosed simultaneously with CHD and NET (Table 1). With respect to time point initial CHD diagnosis, the corrected 1, 3 and 5-year mortality rates following the CHD diagnosis were 32% (7 patients deceased); 50% (10 patients) deceased and 63% (12 patients deceased) (Fig. 1A). When analyzing survival rates, we noted that there was a difference in median and mean values with respect to survival following CHD diagnosis (mean 24 months [SD 25 months] vs median 16 months [iqr 34 months]) and time to CHD diagnosis following diagnosis of cancer (mean 15 months [SD 21 months] and median 3 months [iqr 28 months]) (Table 1) suggesting that these patient parameters don’t follow a Gaussian distribution. This prompted us to plot individual survival rates/time to data lock point of patients against time to CHD diagnosis (Fig. 1B). This allowed us to graphically identify patients from group C. Using the maximal survival rate following CHD diagnosis from patients from group C (17 months) as cut-off allowed us to distinguish between patients from group A and B.

- Group A consists of patients which were diagnosed with a neuroendocrine neoplasm and shortly after with CHD succumbing to the disease within a median time of 5 months. Of note, one patient located in the group A circle, was excluded from the analysis as the patient was still alive and the disease is stable at time of reporting.
- Group B comprised patients which were simultaneously diagnosed with a neuroendocrine tumor and CHD having survived with a median time frame of 4 years.
- Group C included patients who were diagnosed with CHD within a median time frame of 4 years following NET diagnosis and succumbing to disease shortly after the diagnosis within a median time of 4 months (at time of reporting 1 patient was still alive but receiving palliative care).

**Table 1:** Overview of patient characteristics

	**All patients, *n* = 23**	**Group A**^**b**^**, *n* = 4**	**Group B *n* = 12**	**Group C *n* = 6**
**Sex, n (%)**				
Female	7 (30)	1 (25)	4 (33)	2 (33)
Male	16 (70)	3 (75)	8 (67)	4 (67)
**Age at diagnosis, years**				
Mean (SD)	64 (10)	74 (7)	65 (10)*	56 (7)*
Median (iqr; min; max)	65 (15; 45; 80)	76 (9; 64; 80)	70 (15.5; 49; 78)	60 (6; 45; 65)
**Death Certified, n (%)**				
No	8 (35)	0 (0)	6 (50)	1 (17)
Yes	15 (65)	4 (100)	6 (50)	5 (83)
**Death tumor-related, n (%)**				
Yes	15 (100)	4 (100)	6 (100)	5 (100)
**Time-to-death after NET diagnosis, months**^**a**^				
Mean (SD)	15 (21)	3 (2)	4 (6)	47(9)
Median (iqr; min; max)	3 (28; 0; 61.4)	2 (2;1;6)	0.4 (3;0;20)	48 (12;28; 62)
**Time-to-death after diagnosis of CHD, months**^**a**^				
Mean (SD)	24 (25)	7 (6)	50 (18)* +	6 (6)
Median (iqr; min; max)	16 (34; 0; 144)	5 (6;1;16)	49 (26;30;75)	4 (2;0;17)

Patient data derived from SwissNET (*n* = 23)

Fisher exact test < 0.05: * vs group A; ° vs group B, + vs group C

*CHD* Carcinoid heart disease, *iqr* Interquartile range, *min* Minimum, *max* Maximum, *NET* Neuroendocrine tumor, *SD* Standard deviation

^a^Only patients who succumbed to disease were evaluated

^b^One patient was excluded from group A as this patient was still alive and the disease is stable

**Table 2:** Neuroendocrine tumor characteristics

	**All patients, *n* = 23**	**Group A**^**a**^**, *n* = 4**	**Group B *n* = 12**	**Group C *n* = 6**
**Primary tumor site, n (%)**				
Ileum/Jejunum	12 (52)	2 (50)	7 (58)	3 (50)
CUP	8 (35)	2 (50)	5 (42)	2 (33)
Pancreas	1 (4)	0 (0)	0 (0)	1 (17)
Caecum	1 (4)	0 (0)	0 (0)	0 (0)
Atypical lung carcinoid	1 (4)	0 (0)	0 (0)	1 (17)
**Diagnosis, n (%)**				
Neuroendocrine carcinoma	3 (13)	0 (0)	1 (8)	2 (33)
Neuroendocrine tumor	19 (82)	4 (100)	11 (92)	3 (50)
Not known	1 (5)	0 (0)	0 (0)	1 (17)
**Functional tumor, n (%)**				
Yes	20 (87)	3 (75)	10 (83)	6 (100)
Not known	3 (13)	1 (25)	2 (17)	0 (0)
**Type of functional tumor, n (%)**				
Carcinoid	17 (74)	2 (50)	8 (67)	6 (100)
VIPoma	1 (4)	1 (25)	0 (0)	0 (0)
unknown	5 (22)	1 (25)	4 (33)	0 (0)
**Metastasis, n (%)**				
Liver	23 (100)	4 (100)	12 (100)	6 (100)
Other site	21 (91)	2 (50)	11 (92)	6 (100)
Lymph Nodes	12 (52)	0 (0)	9 (75)*	5 (83)*
Lung	2 (9)	0 (0)	1 (8)	1 (17)
Bone	11 (48)	2 (50)	4 (33)	5 (83)
Peritoneum	8 (35)	1 (25)	5 (42)	3 (50)
Other	8 (35)	0 (0)	4 (33)	3 (50)
**Histology, n (%)**^**b**^				
G1	14 (74)	2 (50)	9 (82)	3 (75)
G2	5 (26)	2 (50)	2 (18)	1 (25)

Patient data derived from SwissNET (*n* = 23)

Fisher exact test < 0.05: * vs group A; ° vs group B, + vs group C

*CUP* Cancer of unknown origin, *VIP* Vasoactive peptide

^a^One patient was excluded from group A as this patient was still alive and the disease is stable

^b^Data were not reported for all patients

**Table 3:** Overview of echocardiographic alterations in patients with CHD

	**All patients, *n* = 23**	**Group A**^**a**^**, *n* = 4**	**Group B, *n* = 12**	**Group C, *n* = 6**
**Endocard Fibrosis, n (%)**				
Yes	4 (17)	1 (25)	1 (9)	2 (33)
No	14 (61)	2 (50)	8 (66)	3 (50)
Not known	5 (22)	1 (25)	3 (25)	1 (17)
**Myocardial Decompensation, n (%)**				
Yes	6 (26)	0 (0)	3 (25)	3 (50)
No	14 (61)	3 (75)	8 (66)	2 (33)
Not known	3 (13)	1 (25)	1 (9)	1 (17)
**Pulmonary Valve Stenosis, n (%)**				
Yes	6 (26)	2 (50)	2 (16)	2 (33)
No	13 (57)	1 (25)	7 (58)	4 (67)
Not known	4 (17)	1 (25)	3 (25)	0 (0)
**Pulmonary Valve Insufficiency, n (%)**				
Yes	12 (52)	3 (75)	8 (75)	1 (16)
No	8 (35)	1 (25)	2 (12)	4 (66)
Not known	3 (13)	0 (0)	2 (12)	1 (16)
**Tricuspid Valve Stenosis, n (%)**				
Yes	12 (52)	2 (50)	6 (50)	4 (67)
No	19 (39)	1 (25)	5 (41)	2 (33)
Not known	2 (9)	1 (25)	1 (9)	0 (0)
**Tricuspid Valve Insufficiency, n (%)**				
Yes	22 (96)	4 (100)	12 (100)	5 (83)
No	1 (4)	0 (0)	0 (0)	1 (17)
Not known	0 (0)	0 (0)	0 (0)	0 (0)

Patient data derived from SwissNET (*n* = 23)

No significant difference could be detected using Fisher exact test

^a^One patient was excluded from group A as this patient is still alive and the disease is stable

**Table 4:** Therapies in patients with CHD

	**All patients, *n* = 23**	**Group A**^**a**^**, *n* = 4**	**Group B, *n* = 12**	**Group C, *n* = 6**
**Therapy**^**b,c**^				
Somatostatin analogue therapy, n (%)	22 (96)	4 (100)	11 (92)	6 (100)
Chemotherapy, n (%)	5 (22)	0 (0)	1 (8)	4 (67)
Molecular Therapy, n (%)	6 (26)	0 (0)	4 (33)	2 (33)
Ablative Therapy, n (%)	2 (9)	0 (0)	2 (17)	0 (0)
Peptide receptor radionuclide therapy	13 (57)	1 (25)	7 (58)	5 (83)
Surgery, n (%)	11 (48)	1 (25)	7 (58)	3 (50)

Patient data derived from SwissNET (*n* = 23)

No significant difference could be detected using Fisher exact test

*CHD* Carcinoid heart syndrome

^a^One patient was excluded from group A as this patient was still alive and the disease is stable

^b^Data were not reported for all patients

^c^Some patients received multiple therapies

With respect to survival after CHD diagnosis, there was no difference between groups A and C as all patients succumbed to disease within 18 months post diagnosis (Fig. 1C). For group B, 50% of patients were deceased with a corrected 1, 3 and 5-year mortality rate of 0.0%; 18.2% (2 patients deceased) and 40.0% (4 patients deceased), respectively (Fig. 1C, log-rank test Group A or C vs Group B *p* =  < 0.005). When looking at overall survival rates, patients from group A had a median survival of 9.7 months and all patients succumbed to disease within 18 months after the diagnosis (Fig. 1D). Until the 3-year data-point post NET diagnosis, survival rates were similar between groups B and C (Fig. 1D). However, the survival curves differed significantly at the 5-year data-point, only 40% of patients in group C were still alive in comparison to 72% of patients from group B (log-rank test *p* = 0.03). Baseline characteristics of the whole study population and groups A-C are summarized in Table 1. Median age at diagnosis across the whole population was 65 years (range 45–80 years) and the majority of patients were male (70). The only significant difference with respect to demographics and clinical parameters was the age of patient at initial diagnosis. Patients from group A were significantly older, 76 years (range 64–80) than patients from group B, 70 years (range 49–78, group A vs group B: Fisher exact test < 0.05) and group C, 60 years (range 45–65, group A vs group C: Fisher exact test < 0.05). Ileum/ jejunum were the most frequent primary sites (52%) followed by cancer of unknown primary (CUP) (35%) (Table 2). Other primary tumor sites included pancreas, caecum or atypical lung carcinoma, each diagnosed in one patient. Most patients were diagnosed with a NET (*n* = 19; 82%) which was considered functional in the majority of patients (*n* = 20; 87%) (Table 2). For three patients, it was not reported whether the tumor was functional or not. In most cases, the functional type of tumor was carcinoid (74%) (Table 2). Liver metastases were detected in all patients with CHD and the majority of patients presented with metastases at other sites (91%) as well, mainly in the lymph nodes (52%) and bone (48%). In the majority of patients, the tumor was graded as NET G1 tumor (74%) (Table 1). In group C, two tumors were grade NEC (Table 1), however no difference with respect to survival in comparison to the other four patients with a NET tumor from this group were noted (data not shown). It is worth to mention that our study did grade NET G3 as NEC, which was common practice until the 2019 WHO Classification of Tumors of the digestive system was released [27]. Between groups A – C, no statistically relevant alterations were noted. Echocardiographic alterations found in patients with CHD included: TV insufficiency in 22 patients (96%); TV stenosis in 12 patients (52%); PV insufficiency in 12 patients (52%), PV stenosis in 6 patients (26%), myocardial decompensation in 5 patients (22%) and endocardial fibrosis in 4 patients (17%) (Table 3). Heart pathologies in patients from groups A and C tended to be more pronounced in comparison to patients from group B. Patients from the SwissNet cohort received multiple treatment approaches. Nearly all patients (96%) received somatostatin analogues (Table 4). Other treatments included chemotherapy (5 patients); molecular therapy (6 patients); ablative therapy (1 patient), irradiation (13 patients) and surgery (11 patients) (Table 4). Only 2 patient received valve replacement for CHD.

## Discussion

In this retrospective cohort analysis, we identified 23 patients with CHD out of 108 patients with a NET causing carcinoid syndrome and analyzed their survival outcome. With the availability and increased use of somatostatin analogue therapy the emergence of CHD among patients with a NET causing carcinoid syndrome decreased over the past decades from up 56–66% to 20% which is mirrored by our findings [3, 22, 28]. Survival rates of patients with CHD increased over a time period of 20 years from 18 months in the 1980s to 52 months in the late 1990s while in our cohort, survival rates following initial CHD diagnosis were 24 months ranging from 0 – 144 months.

When excluding patients who received their diagnoses at end stage disease, the only significant parameter between patients with poor (average of 6 months) or long (average of 50 months) survival rates following initial CHD diagnosis was the time to CHD diagnosis following initial NET diagnosis. Similar findings were reported in a recently published study by Fijalkowski et al. noting that CHD may not present with clinical symptoms in the beginning however echocardiographic valve alterations such as tricuspid insufficiency could be detected at an early time point during the course of disease leaving room for surgical interventions and prolonging survival [29]. Interestingly, tricuspid insufficiency was the most reported valve pathology in their and our cohort. Therefore, evaluation of tricuspid insufficiency in patients with carcinoid syndrome could emerge as a new screening recommendation [29].

As the pathomechanisms behind CHD are still incompletely understood, ENETS has founded a taskforce in 2021 to establish new guidelines for diagnosis and treatment options to improve morbidity and mortality in patients with CHD. With respect to treatment options, the European Society for Medical Oncology (ESMO) guidelines recommend somatostatin analogue therapy as standard first-line therapy in patients with carcinoid syndrome as it diminishes tumor progression [30]. In addition to the pharmaceutical interventions, valve replacement should be considered in patients where the tumor has already affected the valves [16]. For disease follow-up, ENETS recommends that tumor markers including chromogranin A, NT-proBNP and 5-HIAA need to be evaluated on a regular basis, annually or semi-annually, depending on the respective levels [20]. To our knowledge, this is not standard practice at every clinical institution. Tumor load is another parameter which needs to be evaluated frequently for instance for the feasibility of surgical downgrading or tumor embolization. We like to highlight that tumor load per se is not well defined as several different terms of tumor load (e.g. tumor slope and tumor growth rate) have been used in the literature [31–35]. In addition, we recommend to use hepatic tumor load as the dysfunction of the liver and the severity of CHD might be directly influenced by this situation of tumor involvement in CHD [36, 37]. Taken together, the SwissNet registry is a powerful data tool to evaluate long-term patient outcomes with observational methods. It is one of the first registries with nationwide patient recruitment and allows to identify and follow up on patients with rare NET driven pathologies including CHD. In our case, we were able to identify 23 patients diagnosed with a NET and CHD due to their different diagnostic time course and survival perspectives. To dive deeper into disease progression for rare NET related pathologies this registry would benefit from a more thorough observational follow-up on biomarkers (NT-proBNP, 5-HIAA and chromogranin A) and clinical data including tumor load and typical carcinoid associated symptoms (abdominal pain, flush and diarrhea).

Therefore, large long-term observational clinical trials are needed to further study markers influencing occurrence, severity, progression and long-term survival of patients with CHD to gain a better understanding of the disease and decrease morbidity and mortality among patients diagnosed with CHD. A prospective cohort study on “Development and Progression of Carcinoid Heart Disease in a Cohort of Adult Patients With Neuroendocrine tumors (CRUSOE-NETs)” plans to enroll 600 patients until 2033 to shed more light on the above open questions [38]. When interpreting these findings, the following limitations of this prospective chart review should be considered: the low total number of patients with CHD in general and per group, the reporting of inconsistent and/ or incomplete data (e.g. tumor load and marker, laboratory parameters, ECG, co-existing cardiac morbidities etc.), lack of access to individual patient-level data, too short follow-up time and data reported differently across the enrolling sites. To our knowledge this has been one of the first nationwide analyses of CHD in patients with NET which should be put into perspective with larger cohorts e.g. data from the ENETS registry.

## Conclusion

This retrospective study of 23 patients with carcinoid heart disease enrolled into the SwissNet database showed that early diagnosis with echocardiographic surveillance of carcinoid heart disease during the course of the NET disease was beneficial to overall survival.

Due to the complex nature of the multifactorial pathology of NET causing carcinoid syndrome and CHD, patients should be followed up in a multi-disciplinary center specialized on NET diagnosis and receive a baseline NT-proBNP profile. If the profile is pathologically elevated, echocardiography with frequent follow-ups to detect and monitor heart involvement especially tricuspidal insufficiency and/ or progression of CHD are recommended as a next disease management in order to manage CHD at an early disease stage with somatostatin analogues or valve replacements, if needed. Further, a thorough laboratory follow-up including markers like NT-proBNP, 5-HIAA and chromogranin A, evaluation of clinical symptoms including abdominal pain, diarrhea, flush, and tumor load is necessary to evaluate disease status and progression.

## Data Availability

The datasets used and/or analyzed during the current study are available from the first and corresponding author on reasonable request.
